# Simultaneous Bilateral Scapular Fractures: A Scoping Review

**DOI:** 10.3390/medicina62040786

**Published:** 2026-04-19

**Authors:** Josip Kocur, Slavko Čičak, Dalibor Kristek, Dalibor Divković, Marko Ivanović, Dino Gregorović, David Matić, Matej Tomić, Sonja Škiljić, Ivana Haršanji Drenjančević, Gordana Kristek

**Affiliations:** 1 Faculty of Medicine, Josip Juraj Strossmayer University of Osijek, J. Huttlera 4, 31000 Osijek, Croatiaskiljicsonja@gmail.com (S.Š.);; 2 Department of Orthopaedics and Traumatology, University Hospital Centre Osijek, 31000 Osijek, Croatia; 3 Department of Pediatric Surgery, University Hospital Centre Osijek, 31000 Osijek, Croatia; 4 Department of Anesthesiology, Resuscitation and ICU, University Hospital Centre Osijek, 31000 Osijek, Croatia

**Keywords:** bilateral scapular fractures, scapula fracture, low-energy trauma, electrical injury, convulsion injury, scoping review, PRISMA-ScR

## Abstract

*Background and Objectives*: Simultaneous bilateral scapular fractures are exceptionally rare injuries and are most commonly associated with high-energy trauma, convulsions, or electrical injury. Their occurrence following low-energy trauma is extremely uncommon. This study aimed to conduct a scoping review of the literature on simultaneous bilateral scapular fractures, with emphasis on demographic characteristics, mechanisms of injury, fracture patterns, treatment strategies, and clinical outcomes. To provide clinical context, the findings are illustrated by a case of a 43-year-old previously healthy recreational athlete who sustained simultaneous bilateral scapular fractures after a low-energy fall directly onto the back. *Materials and Methods*: A scoping review of the literature was conducted in accordance with PRISMA-ScR guidelines using the PubMed/MEDLINE and Scopus databases. Studies reporting simultaneous bilateral scapular fractures were identified and analyzed with respect to demographic characteristics, mechanisms of injury, fracture patterns, treatment modalities, and outcomes. *Results*: Thirty-seven studies published between 1946 and 2025 were included, comprising a total of 43 patients. Most cases resulted from high-energy trauma (41.9%), convulsions (25.6%), or electrical injury (16.3%). Low-energy trauma and spontaneous fractures were rare. The scapular body was the most commonly involved anatomical region. Conservative treatment predominated and was generally associated with favorable functional outcomes, while surgical intervention was reserved for displaced or intra-articular fractures. The illustrative case involved bilateral comminuted extra-articular fractures of the scapular bodies and spines without associated injuries and was managed conservatively, resulting in complete fracture healing and full, painless shoulder range of motion. *Conclusions*: The findings of this scoping review, illustrated by the representative clinical case, indicate that simultaneous bilateral scapular fractures may occur even after low-energy trauma in otherwise healthy individuals. Bilaterality alone should not be interpreted as an independent indication for surgical treatment when fractures are stable and minimally displaced. A high index of clinical suspicion and appropriate radiological evaluation are therefore warranted, particularly in emergency and trauma settings, in order to avoid missed or delayed diagnosis, even in cases with seemingly benign mechanisms of injury.

## 1. Introduction

Scapular fractures are exceptionally rare injuries, accounting for less than 1% of all fractures [1]. Despite the relatively large surface area of the scapula, the low incidence of fractures is likely attributable to its enclosure within a strong muscular envelope and its inherent mobility, which allows partial absorption and dissipation of traumatic energy. These fractures most commonly result from high-energy trauma, particularly motor vehicle collisions and falls from a height, and frequently occur as part of polytrauma [1,2,3,4]. Less commonly, they arise secondary to powerful muscle contractions during convulsions or electrical injuries, in which—besides glenohumeral dislocations—fractures of various parts of the scapula may also occur [1,2,4]. In addition to these mechanisms, scapular fractures may also occur in specific sports-related settings, particularly in activities involving repetitive overhead motion such as volleyball, baseball, and handball, or sudden loading of the shoulder girdle. In such contexts, sport-specific adaptations in range of motion, muscular balance, and scapulothoracic coordination may alter load transmission across the shoulder girdle. From a therapeutic perspective, most scapular fractures are treated conservatively, whereas surgical treatment is generally reserved for selected cases, particularly those with substantial displacement, instability, or intra-articular involvement. Simultaneous bilateral scapular fractures are exceedingly rare and have been described in the literature almost exclusively as a consequence of high-energy trauma, convulsions, or electrical shock [2,3,4,5,6,7,8,9,10,11,12,13,14,15,16,17,18,19,20,21,22,23,24,25,26,27,28]. Due to their rarity, the available literature is limited primarily to case reports and small case series, and current knowledge is largely based on descriptive data.

In this study, we conducted a scoping review of the literature to identify and analyze previously reported cases of bilateral scapular fractures. Particular emphasis was placed on demographic characteristics, mechanisms of injury, fracture patterns, associated injuries, therapeutic approaches and clinical outcomes. In addition, the clinical presentation of this rare injury pattern is illustrated with a representative case of a 43-year-old previously healthy recreational athlete who sustained bilateral scapular fractures following a low-energy fall from standing height directly onto the back. This illustrative case provides clinical context for the findings derived from the literature and highlights that such injuries may occur even after seemingly minor trauma.

## 2. Materials and Methods

A scoping review of the literature was conducted in accordance with the Preferred Reporting Items for Systematic reviews and Meta-Analyses extension for Scoping Reviews (PRISMA-ScR) guidelines, given the rarity of the condition and the heterogeneity of the available data [29]. The PRISMA checklist is available as Supplementary File S1. The aim of the review was to identify and analyze published cases of simultaneous bilateral scapular fractures, with particular emphasis on mechanisms of injury, clinical characteristics, treatment approaches, and reported outcomes.

The literature search was performed using the PubMed/MEDLINE and Scopus electronic databases. The PubMed/MEDLINE search strategy included the following terms: “bilateral” AND (“scapula” OR “scapular”) AND “fracture”, using Boolean operators (AND, OR) to combine keywords. Although Medical Subject Headings (MeSH) were not systematically applied, the selected keywords were chosen to maximize sensitivity for identifying relevant case reports.

The Scopus search was conducted using the following query applied to title, abstract, and keywords fields: TITLE-ABS-KEY (bilateral AND (scapula OR scapular) AND fracture). The Scopus search was limited to studies involving humans. No language restrictions were applied. For studies published in languages other than English, translation was performed to ensure accurate data extraction. All articles published up to 31 December 2025 were considered eligible.

Additional relevant studies were also identified through manual screening of the reference lists of all included articles in order to minimize the risk of missing eligible reports. Grey literature was not systematically searched, which may have limited the identification of non-indexed or unpublished reports. All studies describing simultaneous bilateral scapular fractures in adult or adolescent patients with skeletal maturity were eligible for inclusion. Studies involving skeletally immature pediatric populations, non-simultaneous fractures, reports lacking clear patient data or injury mechanisms, as well as archaeological reports of bilateral scapular fractures, were excluded. Titles and abstracts were screened, followed by full-text assessment of potentially eligible studies. Final study selection was performed according to the predefined inclusion and exclusion criteria. From the included studies, data were extracted regarding patient age and sex, mechanism of injury, fracture location, associated injuries and comorbidities, treatment modality, and reported functional outcomes. The extracted data are presented in a tabular format.

The protocol for this scoping review was prospectively registered with the Open Science Framework (OSF; registration number: q4jhc). No critical appraisal of study quality was performed, in accordance with the methodological framework of scoping reviews and PRISMA-ScR recommendations.

## 3. Results

**Figure 1:**
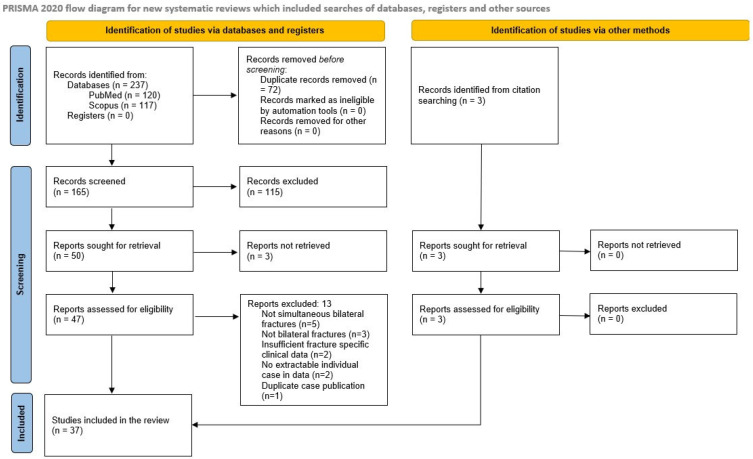
PRISMA-ScR flow diagram of the literature search and study selection process.

The database search of PubMed/MEDLINE and Scopus using the predefined keywords yielded a total of 237 records. After removal of duplicates, 165 records remained for title and abstract screening. Following screening, 50 reports were sought for retrieval. Of these, three reports could not be retrieved, resulting in 47 full-text articles assessed for eligibility from database sources. In addition, three studies were identified through manual screening of the reference lists of the included studies and were also assessed for eligibility. After applying the predefined inclusion and exclusion criteria, 13 studies from the database search were excluded, while no additional exclusions were recorded among studies identified through citation searching. Ultimately, 37 studies were included in the final analysis. The included publications, published between 1946 and 31 December 2025, were predominantly limited to isolated case reports, with only one case series available. In total, 43 patients with simultaneous bilateral scapular fractures were analyzed. The study selection process is illustrated in Figure 1.

The extracted characteristics of the 43 included patients, including demographics, fracture patterns, mechanisms of injury, treatment modalities, and outcomes, are summarized in Table 1. Due to the rarity of the condition, detailed case-by-case reporting was retained despite variability in reporting and frequent missing data (not reported, NR), in order to preserve completeness of the available evidence.

### 3.1. Demographic Characteristics

The analyzed patient population was predominantly male (83.7%, 36/43 patients), while females accounted for 16.3% of cases. Patient age ranged from 17 to 79 years, with a median age of 41 years (interquartile range [IQR], 30–54). The main demographic, etiological, and treatment characteristics of the included patients are summarized in Table 2. Given the relatively small sample size, the reported percentages should be interpreted with caution and are intended to provide a descriptive overview rather than precise estimates.

### 3.2. Anatomical Distribution of Fractures

Fracture location was analyzed according to the anatomical regions of the scapula. The glenoid, neck, body, coracoid, and acromion were considered the main regions, while the scapular spine, superior margin, and superior angle were analyzed as distinct subregions, in accordance with the descriptions provided in the original reports. A total of 86 scapulae were analyzed, with certain fracture patterns involving multiple anatomical regions; therefore, the cumulative percentage exceeds 100%.

The scapular body was the most commonly involved region, affected in 59 of 86 scapulae (68.6%). Fractures of the scapular neck were identified in 17 scapulae (19.8%), and glenoid fractures in 12 scapulae (14.0%). Coracoid process fractures were observed in 5 scapulae (5.8%), acromion fractures in 5 scapulae (5.8%), fractures of the superior margin in 4 scapulae (4.7%), scapular spine fractures in 3 scapulae (3.5%). Fractures of the superior angle were reported in 2 scapulae (2.3%).

### 3.3. Mechanisms of Injury

The most common mechanism of injury was high-energy trauma resulting from motor vehicle collisions or falls from height, accounting for 41.9% (18/43) of all cases. This was followed by convulsions in 25.6% (11/43) and electrical injury in 16.3% (7/43) of cases. Spontaneous fractures accounted for 7.0% (3/43), while low-energy trauma fractures occurring during forced movements against resistance each represented 4.7% (2/43) of cases.

### 3.4. Associated Injuries and Comorbidities

Associated injuries were most commonly observed in fractures resulting from high-energy trauma and primarily involved thoracic injuries, including rib fractures, as well as fractures of the spine or other parts of the upper extremity. Head injuries, pelvic and intra-abdominal organ injuries, and fractures of the lower extremities were also reported. Neurological and metabolic comorbidities were frequently associated with fractures caused by convulsions.

### 3.5. Treatment and Outcomes

Information regarding treatment modality was available for 41 of the 43 patients. Surgical treatment was performed in 8 patients (18.6% of all cases; 19.5% among cases with reported treatment), while 33 patients were managed conservatively.

When analyzed per scapula (n = 86), surgical management was undertaken in 10 scapulae (11.6%), while the remaining scapulae were treated conservatively. In the majority of surgically treated cases (6/8), operative intervention was performed unilaterally despite the presence of bilateral fractures. Functional outcomes were reported in a subset of the included studies. In most cases, good to excellent shoulder ROM was documented. Functional outcomes were inconsistently reported across the included studies and were frequently described qualitatively (e.g., “full ROM” or “good outcome”) without the use of standardized assessment tools. In a substantial proportion of cases, functional outcomes were not reported at all. Given the heterogeneity and incompleteness of the available reports, these findings should be interpreted as descriptive patterns rather than comparative evidence.

Overall, the available evidence remains limited to isolated case reports and small case series, highlighting the rarity of simultaneous bilateral scapular fractures.

## 4. Discussion

### 4.1. Clinical Significance of the Review Findings

The present scoping review analyzed the demographic characteristics, mechanisms of injury, clinical features, and treatment approaches reported in cases of simultaneous bilateral scapular fractures. The findings confirm that the majority of reported bilateral scapular fractures occur in the context of high-energy trauma, convulsions, or electrical injury, whereas low-energy mechanisms are exceedingly rare [1,2,3,4,5,6,7,8,9,10,11,12,13,14,15,16,17,18,19,20,21,22,23,24,25,26,27,28,30,32,33,34,35,37,38]. The illustrative case presented in this study therefore represents an atypical, yet clinically significant, manifestation of this injury pattern. Given the potential for missed diagnosis on standard radiographs and the frequent association with thoracic injuries, early computed tomography (CT) evaluation of the shoulder girdle and thorax should be strongly considered in patients presenting with bilateral shoulder pain after trauma, even when the mechanism appears low-energy.

### 4.2. Historical and Contemporary Context of Bilateral Scapular Fractures

Paleopathological studies suggest that bilateral scapular fractures may have occurred historically. Repeated application of force directly to the back—likely related to forms of corporal punishment commonly practiced at the time—has been proposed as a possible cause [39,40]. Although these findings do not allow for direct clinical comparison, they provide a historical framework indicating that such fractures may occur outside classical high-energy traumatic scenarios.

The first clinical mention of bilateral scapular fractures in modern medical literature was reported by Findlay in 1931, in a series of 23 patients with scapular fractures, although without a detailed description of the injury mechanism or outcomes [41]. The first thoroughly documented case of bilateral scapular fracture, which was also included in our literature review, was published by Heatly in 1946 [5]. In the decades that followed, the literature remained limited to sporadic case reports, predominantly associated with convulsions or electrical injuries, with a total of 13 publications reported by the end of the 20th century [2,3,4,6,7,8,9,10,11,12,13,30].

Over the past 25 years, a greater number of publications have emerged, largely related to high-energy trauma. Among these, the most notable contribution is the study by Tuček et al. published in 2013, which remains the largest reported case series of simultaneous bilateral scapular fractures to date and includes a comprehensive review of the literature available at that time [1,14,15,16,17,18,19,20,21,22,23,24,25,26,27,28,31,33,34,35,36,37,38].

### 4.3. Demographic and Etiological Characteristics in Literature

One of the key findings of our scoping review was the clear predominance of high-energy trauma, convulsions, and electrical injury as the principal causes of bilateral scapular fractures. Notably, previously reported low-energy injuries occurred in elderly individuals, in whom age-related bone fragility may have contributed to fracture occurrence [15,34]. In contrast, our patient was a middle-aged, physically active male without comorbidities, suggesting that low-energy trauma alone—when combined with direct posterior impact and muscular activation—may be sufficient to produce this injury pattern even in the absence of known predisposing factors.

The analyzed cohort demonstrated a marked male predominance (83.7%), with a median age of 41 years, indicating that this injury pattern most commonly affects middle-aged men. This distribution is likely influenced by a combination of exposure to high-risk injury mechanisms and biomechanical factors. Men are more frequently involved in high-energy trauma, physically demanding sports, and occupational activities, and typically possess greater shoulder girdle muscle mass, which may generate higher forces transmitted to the scapula. The wide age range, encompassing both younger and older patients, further emphasizes that bilateral scapular fractures are not confined to a specific age group but are primarily related to the mechanism of injury.

The illustrative case presented here, a previously healthy and physically active middle-aged recreational athlete without comorbidities, aligns well with the dominant demographic profile reported in the literature but differs in terms of injury mechanism, given the absence of high-energy trauma or predisposing medical conditions.

### 4.4. Anatomical Characteristics of the Fractures

Analysis of the anatomical distribution of scapular fractures demonstrates that the scapular body, neck, and glenoid are most commonly involved, whereas marginal and structurally reinforced regions such as the acromion, coracoid process, scapular spine, and superior margin are affected less frequently, except in cases involving specific force vectors or direct impact. This fracture distribution is consistent across different injury mechanisms and supports the assumption that these regions represent the primary zones of force transmission within the scapula. The predominance of scapular body fractures observed in our cohort (68.6%) may be explained by the distribution of tensile and compressive stresses described in biomechanical and finite element models of the scapula.

### 4.5. Potential Biomechanical Mechanisms of Fracture

Biomechanical and finite element studies of scapular models may provide a partial conceptual framework for understanding the observed distribution of fracture localization. Multiple models have demonstrated that, under shoulder loading—whether generated by muscular contraction or external force—the scapula behaves as a curved beam, with simultaneous compressive and tensile stresses developing within the scapular body and neck. As bone tissue is more susceptible to tensile stress, these regions represent biomechanical weak points within the scapula, which may explain their more frequent involvement in bilateral scapular fractures [42,43,44,45,46]. This concept is consistent with the anatomical distribution observed in our analysis.

Different injury mechanisms may substantially influence the distribution and nature of stress within the scapula. In cases of electrical injury, particularly low-voltage alternating current exposure, prolonged and uncontrolled tetanic contractions of the shoulder girdle musculature occur. In contrast, convulsions are characterized by repetitive, and occasionally sustained, episodes of powerful muscle contractions, resulting in cumulative loading of osseous structures. Although a formal subgroup analysis was not feasible due to limited reporting detail, fractures associated with convulsions and electrical injuries appeared to predominantly involve the scapular body and were frequently bilateral, often demonstrating a tendency toward symmetrical distribution. While the illustrative case presented in our study resulted from a low-energy traumatic mechanism, certain similarities in fracture localization may be observed; however, the underlying biomechanical conditions differ, and direct equivalence between these mechanisms cannot be assumed. Simultaneous contraction of muscles such as the trapezius, rhomboids, levator scapulae, serratus anterior, subscapularis, infraspinatus, teres major, and latissimus dorsi can generate substantial shear, torsional, and tensile forces that are transmitted to the scapular body and neck. As the scapula serves as an attachment site for numerous powerful shoulder girdle muscles, such synchronous contractions result in a concentration of mechanical stresses in relatively weaker regions, particularly at the junction between the scapular body and neck [7,8,26]. In addition to these muscle groups, rotator cuff muscles function as dynamic stabilizers of the glenohumeral joint and contribute to force transmission across the scapula. Muscles such as the supraspinatus, infraspinatus, and subscapularis, due to their broad origin from the scapular body, may contribute to the distribution of forces across the scapula. During tetanic contractions, their simultaneous activation with other shoulder girdle muscles may further amplify complex tensile, shear, and torsional forces, promoting mechanical overload of structurally vulnerable regions.

In the illustrative clinical case, the fracture pattern may be biomechanically interpreted as the result of a combination of a direct posterior impact and simultaneous voluntary or reflex activation of the scapular musculature. The direct impact likely led to transmission of mechanical energy through the scapular spine to the scapular body, resulting in bending of the scapula and the development of concurrent compressive and tensile stresses. Concomitant muscular activation may have further increased stress forces within the scapula, which was effectively fixed between the supporting surface and the thoracic cage, thereby contributing to fracture formation despite the low-energy mechanism. These biomechanical considerations are based on indirect evidence and simplified models and should therefore be interpreted as conceptual rather than definitive explanations of fracture mechanisms.

Sports activities involving repetitive overhead motion, particularly volleyball, represent a specific biomechanical model of shoulder-girdle loading. Recurrent serves and spikes expose the shoulder to substantial stresses and may lead to adaptive changes in soft tissues, osseous structures, range of motion, muscular balance, and scapulothoracic coordination. In this context, scapular dyskinesis and strength imbalance have been associated with a higher risk of shoulder pain and injury in volleyball athletes, while studies in adolescent players suggest that sport-specific adaptations of external rotation and glenohumeral biomechanics may begin early and may be more pronounced in attacking players [47,48].

Although the available evidence does not permit formal subgroup analysis, several descriptive patterns emerge from the published cases. High-energy trauma, convulsions, and electrical injury clearly predominate as mechanisms of injury, whereas low-energy mechanisms appear to be distinctly uncommon and, given the small number of reported cases, should therefore be interpreted with caution. In parallel, conservative treatment was the most frequently reported management strategy and was generally used in cases without clear displacement or articular involvement, whereas operative treatment was more often described in selected displaced or intra-articular fractures. However, these patterns should be interpreted cautiously, as the available evidence is based predominantly on isolated case reports with heterogeneous indications, inconsistent reporting, and incomplete functional outcome data. These observations should not be interpreted as evidence-based recommendations but rather as descriptive patterns derived from heterogeneous case reports.

In addition to traumatic and neuromuscular mechanisms, spontaneous bilateral scapular fractures have also been described in the literature, occurring in the absence of significant external trauma. Such fractures are most commonly associated with pathological conditions of the osteoarticular system, including amyloid arthropathy, particularly in patients undergoing long-term hemodialysis [31]. Furthermore, a case of spontaneous bilateral scapular fracture has been reported in the setting of rotator cuff arthropathy associated with diabetes mellitus and documented osteopenia, as well as in elderly osteoporotic individuals following reverse total shoulder arthroplasty [36,38]. Under these circumstances, reduced bone quality, altered postoperative shoulder girdle biomechanics, and chronic muscular loading may result in fractures even under minimal or routine daily activities. These cases indicate that bilateral scapular fractures do not represent an exclusive consequence of high-energy trauma, convulsions, or electrical injury, but may also occur in the context of systemic and degenerative diseases. Given the increasing use of reverse total shoulder arthroplasty, clinicians should maintain a high index of suspicion for scapular stress fractures in patients presenting with atraumatic shoulder pain.

The available evidence suggests that the majority of reported cases were managed conservatively, with favorable functional outcomes when such outcomes were documented. Surgical treatment was reserved for displaced or intra-articular fractures and for cases associated with additional injuries [5,12,15,24,30,33,36,38]. The illustrative case presented in this study is consistent with the observation that the operative management is not necessarily required solely because the fractures are bilateral, particularly when they are stable and without significant displacement. In contrast to the heterogeneity observed in the literature, the illustrative case presented in this study includes standardized functional outcome measures (Constant–Murley and DASH scores), allowing for a more objective assessment. This approach may contribute to improved comparability and highlights the importance of using standardized outcome measures in future reports of similar cases. This is consistent with the general principles of scapular fracture management, where non-operative treatment remains the standard approach in most stable, non-displaced fractures. Even in bilateral cases, conservative management may result in satisfactory functional outcomes when fractures are non-displaced or minimally displaced [49]. The illustrative case is presented to provide clinical context and should be interpreted as complementary rather than as evidence supporting general conclusions.

### 4.6. Clinical Case Illustration

**Figure 2:**
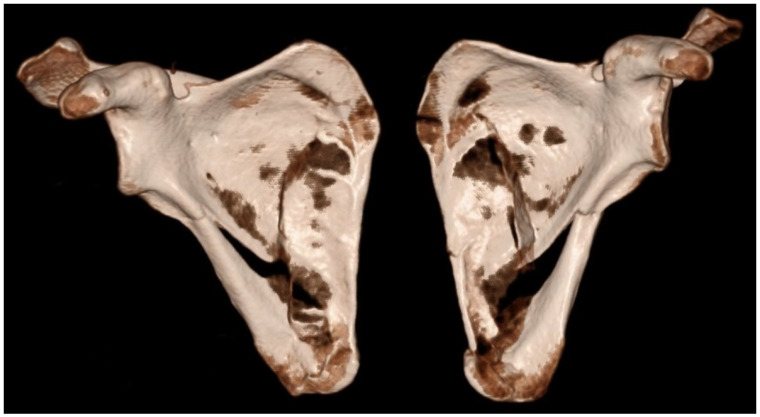
3D CT reconstruction showing bilateral fractures of the scapular body and spine (anteroposterior view).

**Figure 3:**
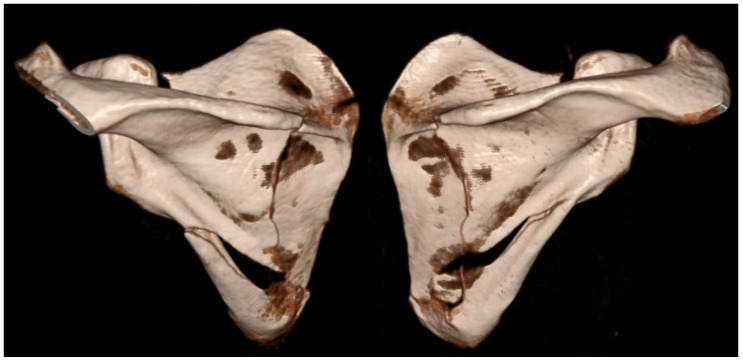
3D CT reconstruction showing bilateral fractures of the scapular body and spine (posteroanterior view).

The following case, which motivated the present review and has been referred to throughout the discussion, is presented in detail below. A 43-year-old previously healthy recreational athlete sustained a low-energy fall from standing height while playing indoor football, landing directly on his upper back. Immediately after the injury, he complained of severe upper back pain and inability to actively elevate his arms. He reported no additional symptoms and denied any previous chronic medical conditions. Clinical examination revealed marked tenderness on palpation over both scapulae, with inability to perform active abduction and forward flexion of the shoulders, as well as pain during passive range-of-motion testing. Neurological examination and peripheral vascular status of the upper extremities were unremarkable. The patient was hemodynamically and cardiopulmonary stable. Initial radiographic evaluation demonstrated bilateral comminuted fractures of the scapular bodies. Subsequent CT confirmed simultaneous bilateral extra-articular fractures involving the scapular bodies and spines, while excluding associated thoracic or intrathoracic organ injuries (Figure 2 and Figure 3).

Given the favorable alignment of the fracture fragments, fracture stability, and absence of associated shoulder joint injuries, conservative management was indicated. Treatment consisted of immobilization of the shoulder girdle with a sling, combined with analgesic therapy and cryotherapy. The patient had no history of chronic medical conditions, and imaging findings were not suggestive of metastatic or metabolic bone disease. After two weeks of immobilization, passive range of motion (ROM) exercises were initiated, followed by gradual progression to active physiotherapy. At the ten-week follow-up, radiographic imaging demonstrated healed fractures of both scapulae. Clinically, the patient exhibited a symmetrical, painless, and full ROM in both shoulders, with return to activities of daily living and sports without limitations (Constant–Murley score: left 98, right 100; Disabilities of the Arm, Shoulder and Hand (DASH) score: left 0, right 0). This illustrative case demonstrates that simultaneous bilateral scapular fracture may occur even after low-energy trauma in otherwise healthy individuals and supports the observations derived from the present scoping review.

### 4.7. Limitations

The limitations of this study include the small number of available cases, the heterogeneity of injury mechanisms, and the variable quality of reporting across individual case reports, together with the inherent limitations of such study designs, including incomplete data and potential publication bias. The interpretation of functional outcomes is limited by heterogeneous reporting, the frequent absence of standardized outcome measures, and the fact that a substantial proportion of cases did not report functional outcomes at all. The literature search was limited to two electronic databases (PubMed/MEDLINE and Scopus), which may have resulted in incomplete identification of all relevant studies, particularly given the rarity of the condition and the predominance of case reports. Given that the available evidence is largely limited to individual case reports, there is an inherent risk of incomplete capture of rare cases, particularly those published in less commonly indexed sources or not indexed in the selected databases. The absence of a systematic grey literature search may have further limited the identification of non-indexed reports.

An additional limitation relates to the biomechanical interpretation of fracture patterns based in part on biomechanical and finite element analyses of the scapula. These analyses rely on simplified static models and cannot fully reproduce the complex dynamic conditions encountered in real-life injury scenarios; therefore, they should be regarded as conceptual support for the clinical findings rather than as direct evidence of fracture mechanisms. Despite these limitations, the present scoping review, supplemented with illustrative clinical case, provides a more comprehensive insight into this uncommon injury pattern and underscores the importance of thorough clinical evaluation even following seemingly low-energy traumatic events.

## 5. Conclusions

Simultaneous bilateral scapular fractures are exceptionally rare and are most commonly associated with high-energy trauma, convulsions, or electrical injury. The findings of this scoping review, together with the illustrative clinical case presented in this study, suggest that this injury pattern may also occur after a low-energy trauma, although such mechanisms appear to be uncommon in the available literature. Most reported cases were managed conservatively, but conclusions regarding the optimal treatment must remain cautious given the low level of evidence and heterogeneous reporting. Management should therefore be individualized according to fracture characteristics and associated injuries rather than the bilateral presentation alone. Increased awareness of this rare injury pattern may help prevent misdiagnosis and ensure timely and appropriate management.

## Figures and Tables

**Table 1:** Summary of published cases of simultaneous bilateral scapular fractures included in the scoping review *.

Author, Year	Age (Years)	Sex	Left Fracture Type	Right Fracture Type	Mechanism of Injury	Associated Injuries	Treatment	Functional Outcome	Comorbidities
Heatly, 1946 [5]	30	M	Body	Body; Neck	MVA (truck struck)	None	R operative, L conservative	Full ROM	NR
Cser, 1976 [6]	35	M	Body	Body	Convulsion	None	Conservative	Full ROM	Epilepsy
Tarquinio, 1979 [7]	41	M	Body; Neck	Body; Neck	Electric shock	Finger burn	Conservative	Full ROM	NR
Beswick, 1982 [8]	43	M	Body; Neck	Body; Neck	Electric shock	None	Conservative	Full ROM	NR
Mathews, 1983 [9]	18	M	Body	Body	Convulsion	None	Conservative	Good ROM	Renal cadaver transplant and extraction, HD
Williamson, 1988 [10]	17	M	Superior margin	Superior margin	MVA	Mild head injury, amnesia	Conservative	Full ROM	None
Wertheimer, 1990 [11]	21	F	Body	Body	Convulsion	None	Conservative	Full ROM	End-stage renal disease, hyperparathyroidism, parathyroidectomy
Dumas, 1992 [2]	46	M	Body; Neck	Body; Neck	Electric shock	None	Conservative	Full ROM	NR
Arenas, 1993 [13]	32	F	Superior margin	Superior margin	Motorcyclist	Right clavicle Fx	Conservative	Full ROM	Steinert myotonic muscular dystrophy
Liaw, 1996 [3]	46	M	Body; Neck	Body; Neck	Electric shock	None	Conservative	Full ROM	NR
Heggland, 1997 [30]	42	M	Glenoid	Glenoid	Weight lifting	Shoulder dislocation, Hill–Sachs lesion	L operative, R conservative	Very good ROM	NR
Cottias, 2000 [12]	33	M	Coracoid	Coracoid	Convulsion (hypoglycemia-induced)	Shoulder dislocation, GT Fx	R operative, L conservative	Full ROM	Diabetes
Kotak, 2000 [4]	51	M	Body	Body	Electric shock	None	Conservative	Full ROM	NR
Yamamoto, 2001 [31]	68	M	Acromion	Acromion	Spontaneous	Humeral head erosions	Conservative	NR	HD-related Amyloid arthropathy
Beaufils, 2004 [32]	47	M	Body	Body	Electric shock	Palm burn	Conservative	Full ROM	NR
Hart, 2004 [33]	24	F	Neck	Neck	MVA	Rib Fx, bil clavicle Fx	R operative, L conservative	Full ROM	NR
Christofi, 2008 [34]	73	M	Body; Neck; Glenoid	Body; Neck; Glenoid	Low-energy fall	None	Conservative	Full ROM	NR
Mavrodontidis, 2008 [35]	19	M	Body	Body	Convulsion	Femoral neck Fx	Conservative	NR	Renal cadaver transplant, hyperparathyroidism, hypertension, parathyreoidectomia
Ejnisman, 2011 [1]	49	M	Body	Body	Skydiving (Forced hyperextension)	None	Conservative	Full ROM	NR
Taneja, 2013 [14]	37	F	Coracoid	Coracoid	Convulsion	Shoulder dislocation, Hill–Sachs lesion	Conservative	Full ROM	Epilepsy
Tuček, 2013 [15]	27	M	Coracoid; Body	Body (open); Glenoid	MVA	Brain contusion, pneumothorax, Fx of the skull, C2, Th IV, Th V, multiple ribs Fx	Conservative	NR	NR
	20	M	Body	Body	Fall from height	Pneumothorax, liver contusion, Fx of the C V- CVII, transverse processes of Th X–LI, pelvis (stable), multiple ribs	Conservative	NR	NR
	78	F	Glenoid	Glenoid	Fall during walk	None	R operative, L conservative	NR	NR
	47	F	Body	Body	MVA	Subdural hematoma, lung contusion, Fx of the left clavicle, multiple ribs, Th V and VI with paraplegia	Conservative	NR	NR
	31	M	Body	Body	Fall from height, paragliding	Rib Fx, lung contusion	Conservative	NR	NR
	31	M	Body; Glenoid	Body; Glenoid	MVA	Left foot Fx and open ankle Fx, perilunar dislocation of the left wrist	Conservative	NR	NR
Karthik, 2014 [36]	61	M	Scapular spine	Scapular spine	Spontaneous	None	L operative, R conservative	Good ROM	Rotator cuff arthropathy, COPD, Diabetes mellitus, osteopenia
Henry, 2013 [16]	42	M	Body	Body	Convulsion	Bil Triceps avulsion	Conservative	NR	NR
Gulbahar, 2015 [17]	77	F	Glenoid	Body	MVA	Bil 1st rib Fx, bil pneumothorax	Conservative	NR	NR
Bartonicek, 2016 [28]	30	M	Body	Body	High-energy trauma	NR	Conservative	NR	NR
Nakata, 2018 [18]	36	M	Superior angle	Superior angle	MVA	None	Conservative	Full ROM	None
Matuszewski, 2019 [19]	59	M	Body	Body	Falling tree	Th IX Fx	Conservative	NR	NR
Biswas, 2019 [37]	66	M	Acromion	Glenoid	Fall from height	Bil GH dislocation (luxation erecta), R Hill–Sachs lesion, massive bil rotator cuff rupture	Conservative	NR	NR
Kim, 2021 [38]	79	M	Acromion	Acromion	Spontaneous	None	Operative	Full ROM	Bilateral rTSA, osteoporosis
Zengui, 2021 [20]	50	M	Body	Body; Spine	Fall from height	None	Conservative	Full ROM	NR
Betten, 2022 [21]	32	M	Body	Body	Convulsion	None	Conservative	Full ROM	Depression
Tobe, 2022 [22]	34	M	Body	Body	Convulsion	None	NR	NR	None
Chouhan, 2023 [23]	45	M	Body; Neck	Body; Neck	Convulsion	None	Conservative	Full ROM	None
Sood, 2023 [24]	54	M	Body	Body	Convulsion	Cerebrovascular thrombosis	Operative	Full ROM	Hypertension
Yadav, 2023 [25]	18	M	Body	Body	MVA	Th V Fx	Conservative	NR	None
Lin, 2025 [26]	58	M	Body	Body	Electric shock	None	NR	NR	None
Aldhaheri, 2026 [27]	29	M	Body	Body	MVA	Lung contusion, pneumothorax, liver and adrenal gland hematoma, Multiple rib Fx, transverse processes of L1–L5 vertebrae, left acetabulum, left pubic bone, and the right iliac bone	Conservative	Full ROM	None
	29	M	Glenoid; Neck	Neck	Struck by marble plates	Pneumohemothorax, lung contusion, liver laceration, multiple rib Fx, mandibular Fx	Conservative	Full ROM	None

* Abbreviations: M—male; F—female; MVA—motor vehicle accident; L—left; R—right; ROM—range of motion; GT—greater tubercle; Fx—fracture; NR—not reported; HD—hemodialysis; COPD—chronic obstructive pulmonary disease: bil—bilateral; rTSA—reverse total shoulder arthroplasty.

**Table 2:** Summary of demographic and clinical characteristics of included patients (n = 43).

Variable	N (%)
Sex	
Male	36 (83.7%)
Female	7 (16.3%)
Mechanism of Injury	
High-energy trauma (MVA, fall from height)	18 (41.9%)
Convulsions	11 (25.6%)
Electrical injury	7 (16.3%)
Spontaneous fractures	3 (7.0%)
Low-energy trauma	2 (4.7%)
Forced movement against resistance	2 (4.7%)
Treatment *	
Surgical (unilateral or bilateral)	8 (18.6%)
Conservative (bilateral)	33 (76.7%)
Not reported	2 (4.7%)

* Treatment data available for 41 patients.

## Data Availability

Data supporting the findings of this study are available within the article.

## Supplementary Materials

The following supporting information can be downloaded at: https://www.mdpi.com/article/10.3390/medicina62040786/s1.

## Abbreviations

The following abbreviations are used in this manuscript:

bil	Bilateral
COPD	Chronic obstructive pulmonary disease
CT	Computed tomography
DASH	Disabilities of the Arm, Shoulder and Hand
Fx	Fracture
GH	Glenohumeral
GT	Greater tubercle
HD	Hemodialysis
IQR	Interquartile range
L	Left
MeSH	Medical Subject Headings
MVA	Motor vehicle accident
NR	Not reported
OSF	Open Science Framework
PRISMA-ScR	Preferred Reporting Items for Systematic Reviews and Meta-Analyses Extension for Scoping Reviews
R	Right
ROM	Range of motion
rTSA	Reverse total shoulder arthroplasty
